# Successful haemostasis for persistent bleeding from a saphenous vein graft needle hole identified by negative contrast echocardiography using intravascular ultrasound: a case report

**DOI:** 10.1093/ehjcr/ytae479

**Published:** 2024-09-05

**Authors:** Shun Nishino, Nehiro Kuriyama, Chiharu Nishino, Yoshisato Shibata

**Affiliations:** Department of Cardiology, Miyazaki Medical Association Hospital Cardiovascular Center, 1173 Arita, Miyazaki 880-2102, Japan; Department of Cardiology, Miyazaki Medical Association Hospital Cardiovascular Center, 1173 Arita, Miyazaki 880-2102, Japan; Department of Cardiology, Miyazaki Medical Association Hospital Cardiovascular Center, 1173 Arita, Miyazaki 880-2102, Japan; Department of Cardiology, Miyazaki Medical Association Hospital Cardiovascular Center, 1173 Arita, Miyazaki 880-2102, Japan

## Case description

**Figure 1:**
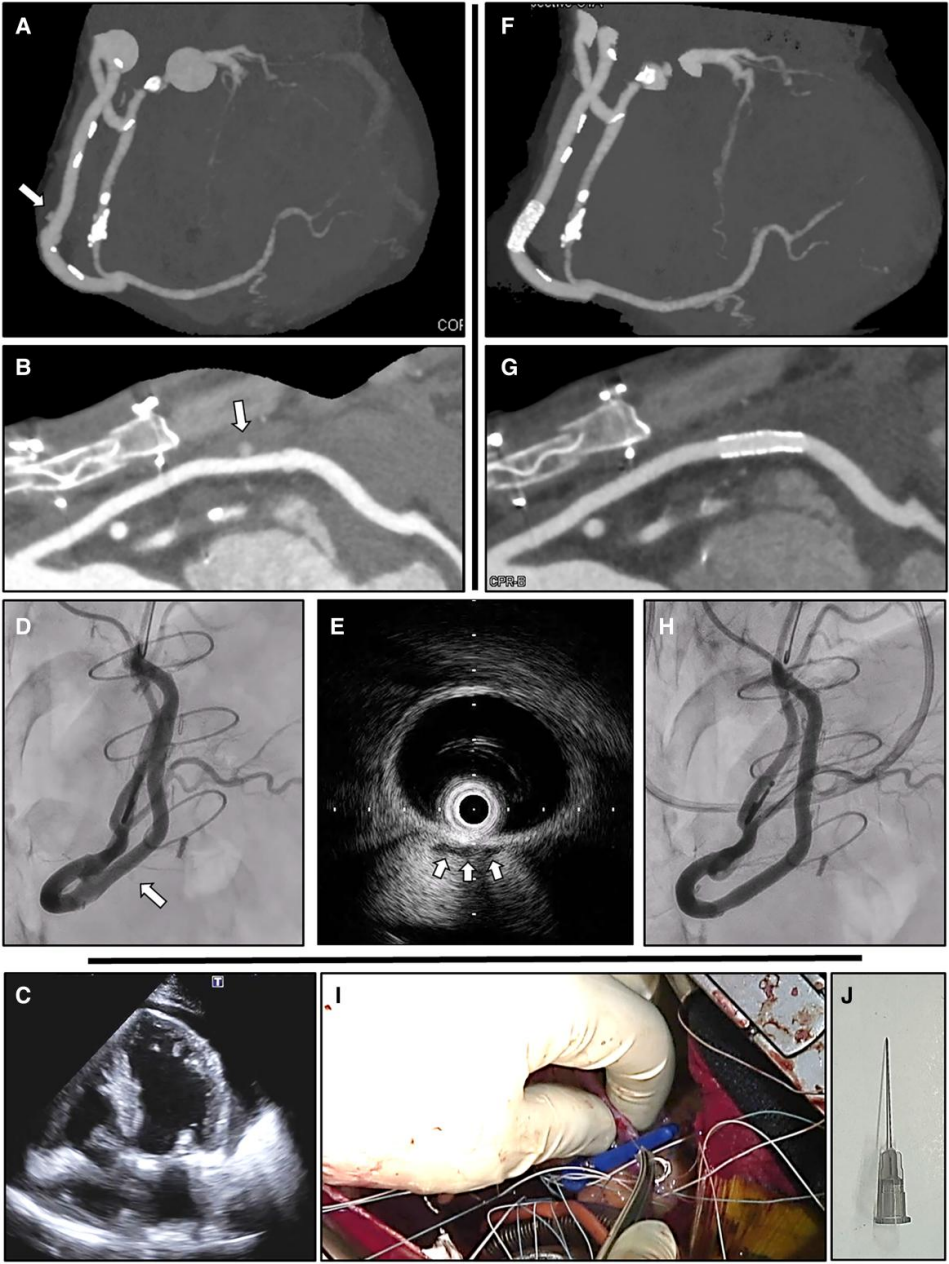
Successful haemostasis for bleeding from a saphenous vein graft needle hole. A computed tomography scan showed contrast extravasation from the centre of the saphenous vein graft between the aorta and the distal right coronary artery (*A* and *B*, arrows). Persistent microbleeding resulted in cardiac tamponade (*C*). It was very difficult to determine the bleeding site on fluoroscopic images (*D*, arrow). After injecting contrast medium while observing intravascular ultrasound images and filling the vessel with negative echo contrast, the bleeding point was identified as the location where a small amount of negative echo contrast was present outside the vessel (*E*, arrows). Successful haemostasis was confirmed after implantation of a covered stent (*F–H*). The bleeding origin was thought to be a needle hole (27G) drilled to deaerate the grafted vein after coronary artery bypass surgery (*I* and *J*).

The patient was a 56-year-old male with severe, symptomatic mitral regurgitation due to mitral valve prolapse and ischaemia in the right coronary artery perfusion area. He underwent simultaneous mitral valve repair and single coronary artery bypass grafting for a single coronary artery (Lipton classification L2-B, malignant course). On the 11th day after cardiac surgery, a computed tomography scan was performed to confirm the patency of the bypass graft, but contrast extravasation was observed from the centre of the saphenous vein graft between the aorta and the distal right coronary artery (*Figure 1A* and *B*, arrows). There was no haemodynamic effect associated with haematogenous pericardial effusion, and anticoagulation therapy was discontinued due to the substantial physical burden of re-thoracotomy. Spontaneous haemostasis was expected thereafter, but microbleeding persisted as of the 17th post-operative day, resulting in cardiac tamponade (*Figure 1C*). Five hundred millilitres of bloody pericardial fluid was drained by pericardiocentesis, and considering the physical burden of the patient, haemostasis was performed percutaneously instead of by re-opening. The bleeding site was very difficult to determine on fluoroscopic images (*Figure 1D*, arrow; Supplementary material online, *Video S1*), but it was successfully identified by applying negative contrast echocardiography to intravascular ultrasound (IVUS) images. After injecting contrast medium while observing IVUS images and filling the vessel with negative echo contrast, the bleeding point was identified as the location where a small amount of negative echo contrast was present outside the vessel (*Figure 1E*, arrows; Supplementary material online, *Video S2*). The stent implantation position was determined by marking the location of a radiopaque marker band corresponding to the transducer of the IVUS catheter. A covered stent (PK papyrus, BIOTRONIK, Germany) was successfully placed to stop the bleeding (*Figure 1F–H*; Supplementary material online, *Video S3*). This is an extremely rare case in which a very small amount of bleeding persisted from a needle hole (27G) intended to deaerate the grafted vein after coronary artery bypass surgery (*Figure 1I* and *J*; Supplementary material online, *Video S4*), leading to cardiac tamponade and necessitating percutaneous haemostasis. Negative contrast echocardiography using IVUS was able to identify the bleeding point that was difficult to visualize fluoroscopically.

## Supplementary Material

ytae479_Supplementary_Data

## Data Availability

All data related to this case report are presented in the published manuscript.

## Supplementary material

**Consent:** The authors confirm that written consent for the submission and publication of this case report, including images and associated text, was obtained from the patient in accordance with the COPE guidance.

**Funding:** None declared.

